# Validation study of GRACE risk scores in indigenous and non-indigenous patients hospitalized with acute coronary syndrome

**DOI:** 10.1186/s12872-015-0138-6

**Published:** 2015-11-16

**Authors:** Pamela J. Bradshaw, Judith M. Katzenellenbogen, Frank M. Sanfilippo, Michael S. T. Hobbs, Peter L. Thompson, Sandra C. Thompson

**Affiliations:** School of Population Health, University of Western Australia, M431, 35 Stirling Highway, Crawley, WA 6009 Australia; Western Australian Centre for Rural Health, University of Western Australia, M706, 35 Stirling Highway, Crawley, WA 6009 Australia; Department of Research, 2nd Floor A Block (Mailbox 26), Sir Charles Gairdner Hospital, Hospital Avenue, Nedlands, 6009 WA Australia

**Keywords:** GRACE risk score, Acute coronary syndrome, Indigenous, Mortality, Aboriginal

## Abstract

**Background:**

Although cardiovascular disease is the major cause of premature death among Indigenous peoples in several advanced economies, no acute coronary syndrome (ACS) risk models have been validated in Indigenous populations. We tested the validity and calibration of three Global Registry of Acute Coronary Events (GRACE) scores among Aboriginal and non-Aboriginal Australians.

**Methods:**

GRACE scores were calculated at admission or discharge using clinical data, with all-cause deaths obtained from data linkage. Scores for GRACE models were validated for; 1) in-hospital death, 2) death within 6 months from admission or 3) death within 6 months of discharge (this also for 1 and 5-years mortality).

**Results:**

Aboriginal patient were younger (62 % aged <55 years versus 15 % non-Aboriginal) and their median GRACE scores lower than non-Aboriginal patients, as was crude mortality at 6 months from admission (6 % vs 10 %) and at 1 and 5 years. After age stratification, risk scores for Aboriginal patients were equivalent or higher, especially among those aged <55 years. There was a trend to more deaths after discharge among Aboriginal patients in each age group, suggesting an age-related under-estimation of risk. The c-statistics for the three GRACE models within both groups were between 0.75 and 0.79.

**Conclusions:**

We demonstrated for the first time that while the discriminatory capacity of GRACE risk scores among Indigenous Australians is good, the models may need re-calibrating to improve risk stratification in this and other Indigenous groups, where age of onset of coronary disease is much younger than among the original reference population.

## Background

Indigenous populations in several advanced economies experience higher rates of coronary heart disease (CHD) and mortality, starting at a younger age than the general population. The disparity, well-documented among Aboriginal and Torres Strait Islander (hereafter Aboriginal) Australians [1], has been reported among indigenous people in countries such as the United States (US), Canada and New Zealand [2–7].

Aboriginal Australians are approximately 3 % of the total population in Australia and 3.8 % of the state population of Western Australian, with around one-third living in the metropolitan area of the state capital, Perth, and 40 % living in ‘remote’ or ‘very remote’ areas during the data collection period [8]. Although this Aboriginal population is relatively young, with a median age of 21 years compared with 37 years for the non-Aboriginal population, CHD is the leading cause of death. CHD was the cause of death for 37 % of Aboriginal people aged 45 to 64 years in 2003–2007, compared with 9 % of non-Aboriginal Australians,[8] and Aboriginal people suffer coronary events at three times the age-standardised rate as other Australians, with the greatest difference among Aboriginal people aged 35–54 years [9, 10]. Aboriginal patients also suffer higher case-fatality after acute myocardial infarction than non-Aboriginals [9, 11, 12].

Among CHD risk models, only the well-validated Framingham Risk Score, used to estimate the 10-year risk for a CHD event, has been tested in indigenous groups [13, 14]. Among remote-dwelling Australian Aboriginal people, the Framingham model substantially under-estimated risk, especially for women and young adults [14].

Due to the high incidence of CHD and poorer outcomes among Aboriginal patients [9–12], accurate estimation of risk at presentation is important to ensure appropriate intervention to preserve optimal myocardial function, and to guide ongoing management. The first Global Registry of Acute Coronary Events (GRACE) score was developed to enable ‘practical and accurate prediction of in-hospital mortality’ for individuals presenting with symptoms of acute coronary syndrome (ACS) [15]. The data for the development of the GRACE ‘in-hospital’ model were gathered from hospitals in 14 countries worldwide. Two additional risk score models have been developed from the registry; to extend to the prediction of risk for death within 6 months of admission, and a score, calculated at discharge, to predict the risk for death within 6 months from discharge [16, 17]. These three models have been tested and validated in studies from several countries and retained good discriminatory capacity for predicting death, including death within one year, and out to five years [18]. A large scale review found that GRACE models performed better than similar ACS risk models. [19] The GRACE risk calculators are available online and are available for downloading to portable devices [20].

Despite the higher risk among several indigenous populations for CVD [2–7] there are no published results validating an ACS risk score in an indigenous population. As the distribution of age, sex, and risk factors contribute to the performance of risk scores among different populations, we tested three GRACE models in a cohort of Western Australian patients admitted for ACS to determine whether these prognostic models performed equally well among Aboriginal and non-Aboriginal Australians.

## Methods

### Study design

This retrospective cohort study used clinical data recorded in medical notes during an admission for ACS, with patients being followed up to five years to determine vital status. These clinical data were linked to public and private hospital administrative data from the Hospital Morbidity Data Collection (HMDC), one of the core administrative datasets in the Western Australian Data Linkage System in which all hospital separations in the state of Western Australia (WA) are collated for individuals under a unique identifier [21]. These linked HMDC records allow person-based analyses, where all admissions for each patient (including inter-hospital transfers) can be identified, with data from the WA Death Registry also linked to these hospital records.

### Sample and case selection

The cohort of ACS patients was selected for this study from two prior studies. The first was a linked data study to validate diagnoses of acute myocardial infarction (AMI) recorded in Western Australian administrative data among patients aged 35–79 years admitted to hospitals in metropolitan Perth (the capital city) in 2003 [22]. Briefly, patients with a World Health Organisation International Classification of Disease (ICD-10 Australian Modification) code for ACS (I20.0 and I21) recorded in the principal or subsequent ‘diagnosis’ field, or with a positive cardiac biomarker and any other ‘cardiac’ or ‘chest pain’ diagnosis were identified (I10-I52, R07). Data were collected from patients’ notes (medical records) for a sample of patients randomly selected within strata (approximately 50 % of ACS cases and 5 % of ‘other’ cardiac cases). The stratified sampling fractions were based on diagnosis ICD codes, admission type (emergency, booked) and cardiac biomarker status [22]. The second study extended the cohort to include all Aboriginal patients admitted to WA hospitals in 2002–2004 and a random sample of rural non-Aboriginal patients admitted in 2003 [23]. These ACS cases, including both metropolitan and rural Aboriginal and non-Aboriginal admissions over the period 2002–2004, were used in the current study. The data collection period coincides with the period of enrolment of patients to the GRACE Registry [18].

Data linkage and extraction for the studies was undertaken by the staff at the Data Linkage Unit at the Health Department of Western Australia.

**Fig. 1:**
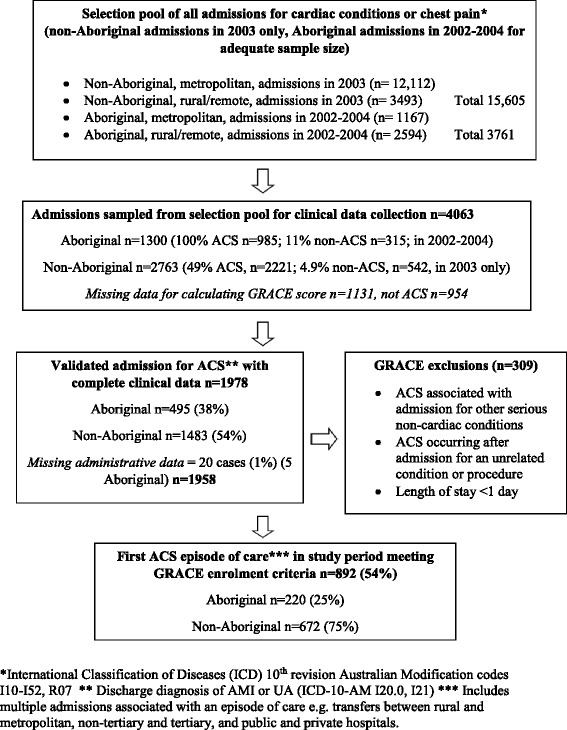
Flow chart showing selection of 892 study cases of acute coronary syndrome admitted to hospitals in Western Australia during 2002–2004

To address under-identification of Aboriginal status in administrative health data, patients were classified as Aboriginal if they were flagged as Aboriginal in any hospital admission in the HMDC data [24]. The index ‘episode of care’ for this analysis was the first admission episode (including transfers between rural and tertiary centres, between other metropolitan hospitals and tertiary centres and between public to private hospitals) within the study period. De-identified cases were selected for the study if they met the published eligibility criteria for enrolment in the GRACE study (based on discharge diagnoses, electrocardiograph [ECG] and biomarker data), excluding those discharged from hospital within one day or who had ACS secondary to another serious condition or a procedure [16]. The selection of cases is shown in Fig. 1.

### Data collection

Clinical information was collected from patient medical records from hospitals throughout WA, including symptoms, investigations, laboratory data, medical history and procedures. The data collection has been described in detail elsewhere [22, 23]. The electrocardiogram (ECG) recordings were coded using the Minnesota system,[25] and classified according to the American Heart Association (AHA) criteria for acute coronary heart disease [26]. Cases with a principal discharge diagnosis of AMI and with AHA ECG classification of ‘diagnostic, ST elevation, or evolving Q waves’ were categorized as ST-elevation myocardial infarction (STEMI). Killip Class categorisation of acute heart failure (HF) (included in the two risk scores calculated on admission) was not recorded for 14 patients, but defaulted to ‘class one’ if they had no history of HF and no in-hospital HF on the index admission.

### Risk score models

The clinical data were used to calculate the three published GRACE scores; calculated (1) on admission for risk of death in-hospital, (2) on admission for risk for death within 6 months of admission, and (3) at discharge for risk for death within 6 months of discharge [20].

All models assign a high weighting to age: for example, under the in-hospital model no points accrue for patients <40 years, but 58 points accrue to those aged 60–69 years, the same as for systolic blood pressure ≤80 mmHg, which is otherwise the highest score for any level of a factor, and greater than cardiac arrest at admission (39 points).

The risk scores from the third model (risk for death within 6-months of hospital discharge) for Aboriginal and non-Aboriginal patients were divided into tertiles for comparison with the tertiles of risk score published from the GRACE study [20]. These stratify patients into low, intermediate and high risk for death according to their diagnosis of STEMI or non-STEMI/unstable angina (UA).

The scores obtained on admission were also categorized by the probability of risk for all-cause death at 6 months estimated from the GRACE study formula,[23] for calibration against the observed proportion of deaths. We used the second model (6 months from admission model) as the number of deaths was higher.

### Study endpoints

The main outcome measures were death in-hospital and 6-month mortality (from both hospital admission and discharge). One-year and five-year mortality after discharge was also measured.

### Statistical analysis

Descriptive analyses were used to characterize the study cohort. Differences between groups defined by age groups (including by more or less than 55 years to account for the younger mean age of Aboriginal patients), sex and Aboriginal and non-Aboriginal status were tested using the chi-squared test for categorical data and the *t*-test for continuous variables.

As the ‘*c*-statistic’ is most frequently used to report the discriminatory capacity of prognostic GRACE risk scores, we plotted the Receiver Operating Characteristic (ROC) curve of GRACE scores against deaths at 30 days, 6 months and 1 and 5 years for the cohort overall, and for Aboriginals and non-Aboriginals separately. Calibration was tested in the ‘admission to 6 months’ model by comparing predicted and observed mortality at 6 months. The Hosmer-Lemeshow ‘p-value’ was obtained from binary logistic regression models as an indicator of the goodness of fit of the three models tested.

All analyses were carried out in IBM SPSS Statistics for Windows, Version 21.0, Armonk, NY: IBM Corp.

### Ethics

The study was approved by the Human Research Ethics Committees at The University of Western Australia, the Western Australian Department of Health, the Western Australian Aboriginal Health Ethics Committee, and the Western Australian Country Health Service.

## Results

**Fig. 2:**
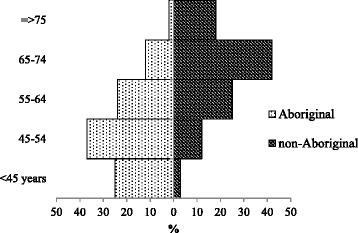
Distribution by age groups among 892 Aboriginal and non-Aboriginal patients discharged with a diagnosis of ACS, Western Australia 2002–2004

Of 892 cases who would have met the GRACE study enrolment criteria, 220 (25 %) were identified as Aboriginal Australians. There was considerable disparity in age, with Aboriginal patients almost 15 years younger on average than non-Aboriginal patients, 136 (62 %) being <55 years of age compared with 99 (15 %) non-Aboriginals (Fig. 2). The demographic and clinical characteristics of the cohort are shown in Table 1.

**Table 1:** Characteristics of the cohort studied, 892 Aboriginal and non-Aboriginal patients discharged from hospital with a diagnosis of acute coronary syndrome, Western Australia 2002–2004

	Non-Aboriginal	Aboriginal	*p*
	*N* = 672	*N* = 220	
Males %	472 (70)	132 (60)	<0.01
Age - mean (SD)	66.4 (9.7)	52.4 (11.4)	<0.001
median (tertile cut points)	68 (63, 73)	52 (47, 57)	
Rural or remote dwelling n (%)	154 (23)	114 (52)	<0.001
*On admission*			
Systolic Blood Pressure – median (mmHg)	140	135	
Heart Rate - median (beats per minute)	75	86	
ST-segment deviation on ECG n (%)	199 (30)	77 (35)	0.15
Elevated initial biomarkers n (%)	241 (36)	125 (57)	<0.001
AMI	172 (64)	99 (79)	
Unstable angina	69 (17)	26 (27)	
Killip class n (%)			
1	533 (79)	185 (84)	
2	92 (14)	22 (10)	
3	44 (6)	13 (6)	
4	3 (<1)	0	
AMI (principal discharge diagnosis)	277 (41)	128 (58)	p < 0.001
STEMI AHA criteria*	102 (37)	30 (24)	
Reperfusion therapy – (N)	64	20	
*Reperfusion therapy for STEMI*	44 (69)	11 (55)	
Thrombolysis (% of reperfusion)	26 (59)	9 (82)	
Primary PCI (% of reperfusion)	18 (41)	2 (8)	
*Reperfusion - ECG with other codes*	20 (31)	9 (45)	
Thrombolysis	12 (60)	9 (100)	
Primary PCI	8 (40)	0	
*History (within 5 years) n (%)*			
AMI	210 (31)	65 (30)	0.86
Heart failure	69 (10)	11 (5)	<0.01
Coronary artery disease	372 (55)	108 (49)	0.15
Chronic renal failure	14 (9)	34 (16)	0.04
Diabetes	228 (34)	124 (56)	<0.001
< 55 years	22 (26)	64 (49)	0.001
≥ 55 years	206 (35)	60 (67)	<0.001
*In-hospital n (% )*			
Heart failure	82 (13)	11 (5)	<0.01
Arrhythmia	80 (12)	8 (4)	<0.001
PCI (excluding primary PCI)	149 (23)	36 (20)	0.22
CABG within 28 days	23 (4)	7 (3)	0.99

*Luepker R, et al. [26]

*AMI* acute myocardial infarction, *STEMI* ST-segment elevation myocardial infarction, *AHA* American Heart Association, *PCI* percutaneous coronary intervention, *CABG* coronary artery bypass graft surgery

### ACS diagnoses

AMI (STEMI and non-STEMI) was the principal discharge diagnosis recorded in the HMDC for 277 (41 %) non-Aboriginal and 128 (58 %) Aboriginal patients (p < 0.001). Using AHA criteria, a total of 132 cases with STEMI (33 %) were identified, with a smaller proportion of Aboriginal patients meeting the criteria for STEMI (Table 1).

Coronary artery reperfusion therapy, either thrombolysis or primary percutaneous coronary intervention (PCI) was undertaken in 89 cases (Table 1), including 55 (61 %) of 90 patients with ST-segment elevation on their first ECG. The other 34 reperfusions were among 92 patients with ischaemic changes (subsequently coded as AHA ‘definite’ or ‘probable’ AMI, with evolving ‘Q-waves’) and 17 patients with a Minnesota suppression codes such as bundle-branch blocks (which suppress the coding of Q waves).

Reperfusion with thrombolysis was more frequent among those presenting initially to a rural hospital (58 %), with only one patient from a rural location undergoing primary PCI. AMI was also more frequently the discharge diagnosis for patients from rural locations (54 %) than from the Perth metropolitan area (40 %, p < 0.001).

### Mortality

Total deaths in hospital, at 6 months (from admission and from discharge), and at one and five years were 39 (4 %), 83 (9 %), 46 (6 %), 80 (13 %) and 232 (30 %) respectively.

Total mortality at each time point favoured Aboriginal patients (Table 2). After age stratification, mortality was similar for both groups for those aged 55 years or more, but was higher among Aboriginal patients <55 years of age from 6 months after hospital discharge. However, the numbers of death were too low (except at 5 years) to show statistical difference.

**Table 2:** GRACE scores from three models, and all-cause mortality, among patients discharged with a diagnosis of acute coronary syndrome for Aboriginal (*n* = 220) and non-Aboriginal (*n* = 672) patients overall and for age groups, Western Australia 2002–2004

	Non-Aboriginal	Aboriginal	Non-Aboriginal	Aboriginal	‘p’ (mean)
	Median (min, max)		Mean (SD)		
GRACE Score at admission for death in-hospital					
All	115 (34, 264)	100 (33, 227)	181.0 (25.8)	105.2 (38.9)	<0.001
< 55 years	75 (34, 163)	88 (33, 73)	117.3 (34.7)	90.2 (32.1)	0.03
≥ 55 years	118 (54, 212)	119 (59, 227)	122.5 (22.6)	126.8 (37.7)	0.26
*Death in-hospital n (%)*	*35 (5)*	4 (2)*			
*< 55 years*	2 (2)*	1 (<1)*			
*≥ 55 years*	*33 (6)*	3 (3)*			
GRACE Score at admission for 6-month mortality					
All	104 (26, 219)	83 (24, 184)	103.4 (29.5)	86.6 (33.9)	<0.001
< 55 years	63 (26, 125)	67 (24, 141)	64.9 (20.4)	70.4 (26.3)	0.11
≥ 55 years	106 (50, 176)	105 (54, 184	108.9 (26.3)	110.1 (29.6)	0.68
*6-month mortality n (%)*	*70 (10)*	*13 (6)*			*0.03*
*< 55 years*	2 (2)*	3 (2)*			*-*
*≥ 55 years*	*68 (12)*	*10 (11)*			*0.53*
GRACE Score at discharge for 6-month mortality					
All	109 (28,194)	89 (20,182)	108.8 (29.5)	93.0 (31.6)	<0.001
< 55 years	65 (28,103)	77 (20, 138)	68.1 (17.9)	77.0 (24.1)	0.004
≥ 55 years,	112 (54, 194)	116 (68, 182)	114.6 (26.1)	116.0 (26.5)	0.64
*6-month mortality n(%)*	*36 (6)*	*10 (5)*			*0.35*
< 55 years	0 (0)*	2 (<2)*			*-*
≥ 55 years	*36 (7)*	*8 (9)*			*0.32*
*One-year mortality n(%)*	*66 (10)*	*14 (7)*			*0.06*
< 55 years	0 (0)*	2 (2)*			*-*
≥ 55 years	*66 (12)*	*12 (14)*			*0.60*
*Five-year mortality n(%)*	*181 (28)*	*51 (24)*			*0.10*
< 55 years	*4 (5)*	*21 (16)*			*0.01*
≥ 55 years	*177 (32)*	*30 (34)*			*0.36*

*less than 5 cases

### GRACE scores

The mean and median scores for each of the three GRACE models (death in-hospital, from admission to 6 months, and from discharge to 6 months) were lower among Aboriginal patients (Table 2). The low scores were related to the relative youth of the Aboriginal patients, 16 % of whom did not accrue any points for age (<40 years). Additionally, only 7 %, compared with 42 % of the non-Aboriginal group, were ≥70 years, an attribute which adds 73 points to the score (in the discharge to 6 months model).

The age disparity was such that, when stratified into broad age groups (<55 and 55–79 years), GRACE scores for Aboriginal patients were equivalent or higher than those of non-Aboriginals, more noticeably in the younger group (Table 2).

### Distribution of scores

**Fig. 3:**
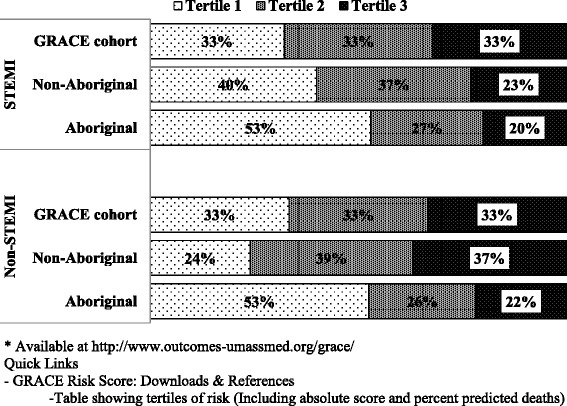
Percentage of patients in tertiles of GRACE risk score for ‘mortality to 6-month post-discharge’ in the GRACE Study cohort*, and for Aboriginal and non-Aboriginal people in our study

Using the ‘6 months post-discharge’ model to compare the distribution of scores with those published for the original GRACE Study cohort [20], the cut points for tertiles of risk score (0.33, 0.66) were lower for Aboriginal than non-Aboriginal patients, and lower than those derived from the GRACE Study population. This was so for STEMI (0.33 corresponds to scores of 85 (Aboriginal), 95 (non-Aboriginal), 99 (GRACE), respectively, and 0.66 to score of 113, 123 and 127, respectively) and non-STEMI/UA (0.33 = 77, 96, 88 and 0.66 = 104, 121 and 118 respectively). Applying the GRACE study tertiles, >50 % of Aboriginal patients would be considered low risk for death within 6 months of discharge for both STEMI and non-STEMI/UA (Fig. 3). For non-Aboriginal patients the variations (more at low risk after STEMI, fewer after non-STEMI/UA) were less marked. The numbers of deaths in each subgroup (tertiles x Aboriginal or non-Aboriginal x AMI or non-STEMI/UA) were too few to make any comparisons of associated mortality.

### Association between GRACE score and outcomes

**Fig. 4:**
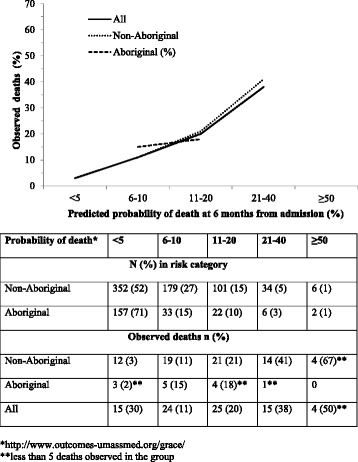
Observed mortality at 6 months from admission among a cohort of patients admitted with ACS including Aboriginal (n = 13 deaths) and non-Aboriginal patients (n = 70 deaths) by the probability of death indicated by the GRACE score*

The ‘admission to 6 month’ model (the highest number of end-points) was well calibrated overall, with good correlation between the predicted probability of 6-month all-cause mortality and observed deaths (Fig. 4). The fit was best for non-Aboriginals, and this resulted in good overall fit, as non-Aboriginals were 75 % of the cohort. For Aboriginal people, however, the association was less convincing, with few deaths among the 70 % in the lowest risk category (<5 % risk), and very few patients in the higher risk groups (>20 % risk). Observed deaths for the 5 Aboriginal patients estimated to be at 6-10 % risk of death to 6 months was 15 %, suggesting under-estimation of risk.

There was little difference in the discriminatory capacity of the GRACE models in both Aboriginal and non-Aboriginal groups, with the *c*-statistics ranging from 0.75 to 0.79. However, there were low numbers of deaths and broader confidence intervals throughout for the Aboriginal cohort (Table 3). Each of the three risk score models was found to demonstrate ‘goodness of fit’ for both groups, with Hosmer-Lemeshow ‘p’-values exceeding 0.05 (Table 3).

**Table 3:** C-statistics* and 95 % CI for three GRACE models for prediction of death at different time points for Aboriginal and non-Aboriginal ACS patients

	c-statistic (95 % CI)			
Group (H-L p-value)*	In-hospital	6 months	One year	Five years
GRACE score on admission : death in-hospital				
Aboriginal	0.76			
(0.63)	(0.62–0.91)			
Non-Aboriginal	0.76			
(0.58)	(0.68–0.84)			
GRACE score on admission : death within 6 months of admission				
Aboriginal		0.76	0.78	0.77
(0.10)		(0.62–0.89)	(0.65–0.91)	(0.70–0.85)
Non-Aboriginal		0.79	0.76	0.75
(0.75)		(0.74–0.84)	(0.71–0.82)	(0.71–0.80)
GRACE score at discharge : death within 6 months of discharge				
Aboriginal	-	0.75	0.77	0.76
(0.30)		(0.56–0.94)	(0.63–0.92)	(0.68–0.84)
Non-Aboriginal	-	0.77	0.78	0.76
(0.87)		(0.71–0.84)	(0.72–0.83)	(0.72–0.80)

* H-L = Hosmer-Lemeshow χ2 p-value

## Discussion

The GRACE risk scores for death in-hospital and to 6 months (from both admission and discharge) have proven reliable in identifying high-risk cases among patients presenting with symptoms of an ACS [18]. Testing three of the models in this population demonstrated that the models retain their prognostic capacity to discriminate between patients at different levels of risk for all-cause mortality after an admission for ACS, confirmed by *c*-statistics of 0.75 or more for both Aboriginal and non-Aboriginal patients at time points to five years.

However, in this cohort, selected ‘post-hoc’ to conform to the GRACE study criteria, crude mortality at each time point was lower among the Aboriginal patients, a finding at odds with the reported excess CVD mortality [1]. Such findings have been reported from studies of outcomes of AMI, where crude case-fatality after AMI for Aboriginal and non-Aboriginal patients was similar, but age-specific rate ratios for Aboriginal patients considerably higher, especially in younger age-groups [11, 12]. The Aboriginal patients in our study may have been relatively low-risk, with a smaller proportion having a diagnosis of STEMI (although more had an AMI diagnosis overall) or suffering in-hospital complications of heart failure or arrhythmia. We had too few deaths to reliably estimate any age-specific outcomes, but stratifying by age <55 years or 55 years or more, there was a trend to more deaths among young Aboriginal people after hospital discharge.

On the small numbers of deaths from admission to 6 months there was an indication of greater than predicted mortality among the ‘low-risk’ Aboriginal patients, and the possibility of underestimation of risk is supported by the equal or higher mean GRACE scores for both younger and older Aboriginal people after age stratification. Other than less likelihood of a history of heart failure, which contributed to lower scores for Aboriginal patients, the other elements of the GRACE scores were equivalent or attracted a higher score for Aboriginal patients. As these factors do not make as significant a contribution to the score as age, the possibility is that the dominance of age in the models contributes to an underestimation of risk, with weightings being less applicable to Aboriginal Australians.

While lacking the power to demonstrate any systematic under-estimation of risk for the GRACE models, there is some support for the caution from the GRACE investigators that the risk models may not perform as well in populations that differ substantially from the population in which the models were developed [17]. Although the GRACE risk models were developed using data from patients from over a dozen countries, minority groups may differ considerably from the study cohort. This is evident for the distribution of age in the Australian Aboriginals admitted for ACS; the mean and median ages in both the GRACE development and validation cohorts were 65 and 66 years respectively [17] ,a year or two younger than the non-Aboriginals in this study (66.4 and 68 years), but more than a decade older than Aboriginal patients at 52.4 and 52 years.

The GRACE Investigators also cautioned that socio-economic status had not been included in the construction of the models, and may well contribute to variability in risk [17]. As Aboriginal Australians and other indigenous minorities within advanced economies are among the most socially disadvantaged [27], the potential for confounding is obvious. However, when tested among patients with AMI in a Canadian study, the GRACE score was found to be a ‘robust, well-calibrated, and accurate risk-stratification tool’ both within and across socioeconomic strata [28]. If the GRACE score performed well in predicting 6-month all-cause mortality among patients hospitalized with AMI, regardless of socio-economic status, the likelihood of confounding by socio-economic status is reduced. Other factors (including age) are likely to influence the capacity of the score to accurately predict the risk for death in Aboriginal patients.

The excessive reduction in Australian Aboriginal life expectancy (the mortality gap), with early CVD being the largest contributor, is a reflection of accrual of lifetime cardiovascular risk. ‘Accrued risk’ among Aboriginal people aged to 50 years was reported to be equivalent to that of Framingham Study participants aged to 70 years for men, and to 80 years for women [29]. Other indigenous populations in which high prevalence of CVD risk factors are reported [2, 4, 5, 7, 30, 31], and in which CVD is manifest at a younger age, include American Indians and Alaskan Natives, Canadian First Nations and Inuit peoples, and the Māori of New Zealand [2–7]. The increased rates of CVD reported for some ethnic groups, associated with changes in lifestyle with immigration, [31–34] are also likely to have distributions of risk that differ significantly from the general population. Opportunities to test the GRACE models in such populations would lead to a better understanding of risk stratification for management of ACS and, plausibly, better outcomes for minority groups.

### Study limitations

Missing data for calculating the GRACE scores (e.g. initial enzyme levels and serum creatinine, ECG, medical history not recorded on data collection form) reduced the ACS cases available for both groups. Despite sampling to capture all ACS admissions among Aboriginal people in the state population across three years only 220 cases had complete data and met the criteria for this study. In addition case-fatality was low for Aboriginal patients, with only four in-hospital deaths and 10 further deaths within six months of discharge, while the rate was as predicted for the non-Aboriginal population. The resulting lack of power left us unable to undertake any analyses that might improve risk stratification among Aboriginal patients, and provide more useful estimates of low, medium and high risk in this population.

The diagnosis of AMI, ACS or unstable angina was that recorded at discharge, rather than on admission as for patients in the GRACE studies. The discharge diagnosis reflects medical decision-making using all the clinical data collected during the admission. For instance, positive biomarkers were not always associated with a discharge diagnosis of AMI, suggesting other clinical data had been taken into consideration.

### Significance

This is the first study to undertake a validation of a widely-used ACS risk stratification model in an Indigenous Minority population. Australian Aboriginals are around 3 % of Australia’s population while 1.7 % of the population of the United States identified as American Indian and Alaska Native, either alone or in combination with one or more other races in 2010 [35], and 4.3 % of Canadians reported an Aboriginal identity in 2011 [36]. Māori are a larger Indigenous Minority, being 15.4 % of the New Zealand population in 2012 [37]. The small size of these populations means that studies are burdened with comparatively low numbers, but the importance of the disparity in morbidity and mortality requires that attention is paid to information that may lead to improvements in disease prevention, detection, and management.

Clinical registries provide the opportunity to understand the risks for and the presentation, management and outcomes of admission of cardiovascular disease. Every effort should be made to ensure Indigenous Minorities are fully represented in such projects.

## Conclusions

The GRACE risk models are useful tools for predicting the risk for in-hospital deaths and all-cause mortality at six months among patients with ACS, but may underestimate risk among Australian Aboriginals who differ significantly from the general population in the age distribution of ACS. If this is the case in other Indigenous or minority populations then re-calibration of the models is necessary to provide more accurate prognostic tools to guide intervention and ongoing management.

## Abbreviations

ACS: Acute coronary syndrome
AHA: American Heart Association
CAD: Coronary heart disease
CVD: Cardiovascular disease
ECG: Electrocardiograph
GRACE: Global Registry of Acute Coronary Events
HF: Heart failure
HMDC: Hospital Morbidity Data Collection
ICD-10-AM: International Classification of Disease 10th edition Australian Modification
PCI: Percutaneous coronary intervention
ROC: Receiver Operating Characteristic
STEMI: ST-elevation myocardial infarction
UA: Unstable angina
US: United States
WA: Western Australia
